# The Effect of Fatal Carbon Monoxide Poisoning on the Surface Charge of Blood Cells

**DOI:** 10.1007/s00232-013-9591-2

**Published:** 2013-08-30

**Authors:** Michał Szeremeta, Aneta D. Petelska, Joanna Kotyńska, Anna Niemcunowicz-Janica, Zbigniew A. Figaszewski

**Affiliations:** 1 Department of Forensic Medicine, Medical University of Bialystok, Waszyngtona St. 13, 15-230 Bialystok, Poland; 2 Institute of Chemistry, University in Bialystok, Al. J. Pilsudskiego 11/4, 15-443 Bialystok, Poland; 3 Laboratory of Electrochemical Power Sources, Faculty of Chemistry, University of Warsaw, Pasteur St. 1, 02-093 Warsaw, Poland

**Keywords:** Fatal carbon monoxide poisoning, Surface charge, pH measurement, Erythrocyte, Thrombocyte

## Abstract

The objective of this investigation was to evaluate postmortem changes of electric charge of human erythrocytes and thrombocytes after fatal carbon monoxide (CO) poisoning. The surface charge density values were determined on the basis of the electrophoretic mobility measurements of the cells carried out at various pH values of electrolyte solution. The surface charge of erythrocyte membranes after fatal CO poisoning as well as after sudden unexpected death increased compared to the control group in the whole range of experimental pH values. Also, a slight shift of the isoelectric point of erythrocyte membranes to high pH values was observed. The surface charge of thrombocyte membranes after fatal CO poisoning decreased at low pH compared to the control group. However, at high pH, the values increased compared to the control group. The isoelectric point of thrombocyte membranes after fatal CO poisoning was considerably shifted toward low pH values compared to the control group. The observed changes are probably connected with the destruction of blood cell structure.

## Introduction

In biological systems, carbon monoxide (CO) is a gaseous second messenger and arises during the oxidative catabolism of heme by the heme oxygenase enzymes. Many biological functions of heme oxygenase, such as regulation of vessel tone, smooth muscle cell proliferation, neurotransmission, platelet aggregation and anti-inflammatory and antiapoptotic effects, have been attributed to its enzymatic product, CO (Bilban et al. 2008).

In physiological conditions heme oxygenases catabolize heme into three products: CO, biliverdin and free iron (Ryter et al. 2006). Heme-derived CO has been proven to modulate neuronally mediated activities, acting as an important neuromodulator, and participates in the regulation of diverse cellular functions, including apoptosis of erythrocytes (Johnson and Johnson 2000). Endogenous CO has a clear role in the apoptosis of erythrocytes, involving cell shrinkage and cell membrane scrambling with phosphatidylserine exposure at the cell surface (Lang et al. 2008).

CO comes from both natural and manufactured sources, the most common being inhaled in the household environment. CO is produced whenever organic materials are burned with an inadequate supply of oxygen necessary to produce complete combustion. Sources of CO poisoning also include motor vehicle exhaust fumes, poorly functioning heating systems and inhaled smoke (Satran et al. 2005).

Exogenous CO can arrest cellular respiration. Immediately after inhalation, the molecule diffuses into the blood of the pulmonary capillaries, crossing the alveolocapillary membrane. The most clear-cut mechanism by which CO toxicity occurs is competitive binding to the hemoglobin heme groups (Rodkey et al. 1974; Guy et al. 1971). This effect is magnified by the allosteric properties of the hemoglobin molecule. Its tetrameric structure undergoes a conformational change when CO is bound at one of the four heme sites, with a resulting increase in the affinity of the remaining heme groups for oxygen (Rodkey et al. 1974; Pace et al. 1950).

Excluding deaths during fires, there are approximately 2,700 deaths caused by CO annually in the United States (Ernst and Zibrak 1998). Approximately 2,000 of these are suicides and 700 are accidents. In France, the annual incidence of unintentional CO poisoning has been estimated at round 18 per 100,000, with a mortality rate of around 200 per year (Gajdos et al. 1991). In northeast Poland, the accidental number of deaths due to CO poisoning is 21 each year (219 of a total of 4,615 autopsies performed in the Department of Forensic Medicine, University in Bialystok, in the years 1998–2008) (Wardaszka et al. 2009).

The chemical and biochemical features of endogenous CO have been well characterized. Due to the lack of literature data concerning the influence of exogenous CO on the electrical properties of biological membranes, we examined changes in the surface charge of blood cells after fatal CO poisoning. This work continues a systematic study of the electrical properties of human erythrocyte and thrombocyte membranes realized by Figaszewski and coworkers (Petelska et al. 2012; Kotyńska et al. 2012). The experiment was performed using a microelectrophoresis method, which is one of the basic analytical tools for biological studies. The electrophoretic mobility measurements were done over a pH range of 2–11. The obtained results in our opinion can help in both the interpretation as well as understanding of the processes that take place on biological membrane surfaces after fatal CO poisoning.

## Materials and Methods

Approval for this study was granted by the Ethics Review Board of the Medical University of Bialystok (no. R-I-002/533/2010). Blood (pH ~6.8) was obtained from sober individuals during autopsies made at the Forensic Medicine Department at the Medical University of Bialystok in the year 2010. The examination was based on 10 selective fatal CO poisonings in fire (six men and four women, mean age 43.4 years, range 23–63, concentration of carboxyhemoglobin >70.81 %) autopsied in the year 2010–2011. Blood was routinely obtained from the femoral vein, put into chemically and biologically clean glass containers and donated to the Department of Electrochemistry at the University of Bialystok. The donated samples were comparatively analyzed with control samples taken from live individuals from the Blood-Service Centre in Bialystok. The carboxyhemoglobin level was determined using a gas chromatography-flame ionization detector.

### Preparation of Erythrocytes from Blood

Erythrocytes were isolated from 2 ml of liquid whole blood by centrifugation at 900×*g* for 8 min at room temperature. The supernatant thrombocyte-rich plasma was removed and saved for subsequent processing, while the erythrocytes were washed three times with isotonic saline (0.9 % NaCl) at 3,000×*g* for 15 min. After the final wash, the erythrocyte pellet was resuspended in isotonic saline for electrophoretic measurement.

### Preparation of Thrombocytes from Plasma

Thrombocyte-rich plasma was centrifuged at 4,000×*g* for 8 min. The supernatant plasma was removed and discarded. The thrombocyte pellet was washed three times with isotonic saline by centrifugation at 3,000×*g* for 15 min. After the final wash, thrombocytes were resuspended in isotonic saline for electrophoretic measurement.

All solutions and cleaning procedures were performed with water purified using a Milli-Qll system (18.2; Millipore, Billerica, MA).

### Microelectrophoretic Mobility Measurements

The electrophoretic mobility of erythrocyte or thrombocyte vesicles in suspension was measured using laser Doppler velocimetry and a Zetasizer Nano ZS (Malvern Instruments, Malvern, UK) apparatus. Measurements were carried out as a function of pH. Cell membranes were suspended in NaCl solution and titrated to the desired pH using HCl or NaOH. The reported values represent the average of at least six measurements performed at a given pH.

From electrophoretic mobility measurements the surface charge density was determined using Eq. 1 (Alexander and Johnson 1949)

1 $$ \delta = \frac{\eta \cdot u}{d} $$

where *η* is the viscosity of the solution, *u* is the electrophoretic mobility and *d* is the diffuse layer thickness.

The diffuse layer thickness (Barrow 1996) was determined from the formula

2 $$ d = \sqrt {\frac{{\varepsilon \cdot \varepsilon_{0} \cdot R \cdot T}}{{2 \cdot F^{2} \cdot I}}} $$

where *R* is the gas constant, *T* is temperature, *F* is the Faraday number, *I* is the ionic strength of 0.9 % NaCl and εε_0_ is the permeability of the electric medium.

## Results and Discussion

The electrophoretic mobility of erythrocytes or thrombocytes in suspension was measured as a function of pH. Cells were suspended in NaCl solution and titrated to desired pH using HCl or NaOH. Electrophoretic mobility values were converted to surface charge density using Eq. 1 presented in section “Materials and Methods.”

**Fig. 1:**
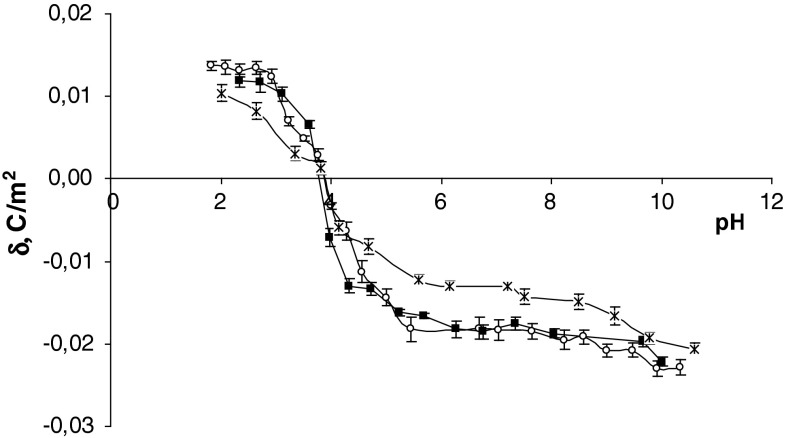
Surface charge density of erythrocytes versus pH of electrolyte solution (*times* control, *open circle* sudden unexpected death, *filled square* fatal CO poisoning)

The surface charge densities of the control, sudden unexpected death and fatal CO poisoning erythrocytes are plotted as a function of pH in Fig. 1. In acid solution, an increase in the positive charge of erythrocyte membranes after fatal CO poisoning in comparison to control erythrocytes was observed. In basic solutions an increase in negative charge after fatal CO poisoning in comparison to control erythrocytes and a small shift of the isoelectric point of the membrane to high pH values were observed. The membrane surface charge density values in fatal CO poisoning presented here were compared with the membrane surface charge density values after sudden unexpected death obtained by us previously (Kotyńska et al. 2012). As can be seen, the values shown in Fig. 1 are similar in both cases.

**Fig. 2:**
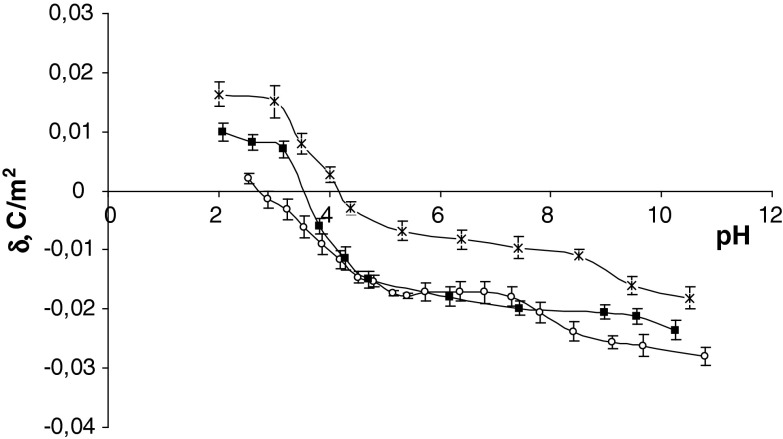
Surface charge density of thrombocytes versus pH of electrolyte solution (*times* control, *open circle* sudden unexpected death, *filled square* fatal CO poisoning)

The surface charge densities of the control, sudden unexpected death and fatal CO poisoning thrombocytes are plotted as a function of pH in Fig. 2. Fatal CO poisoning causes a decrease in the positive charge of the membrane in an acid solution compared with control membrane. In a basic solution it causes an increase in negative charge of the thrombocyte membrane compared with control membrane. Also, a shift of the isoelectric point of the membrane to low pH values was observed. As can be seen from Fig. 2, fatal CO poisoning and sudden unexpected death surface charge density values are similar at pH > 4 but diverge in the low pH range.

The isoelectric point and surface charge density values for human erythrocytes and thrombocytes determined using electrophoresis are presented in Tables 1 and 2, respectively. Data are expressed as mean ± standard deviation. These data were analyzed using standard statistical analysis.

**Table 1:** Surface charge density and isoelectric point values for human erythrocytes (control, sudden unexpected death and fatal CO poisoning)

Groups	Isoelectric point	Surface charge density (10^−2^ C/m^2^)	
		At low pH values	At high pH values
Control (Kotyńska et al. 2012)	3.72	1.039 ± 0.103	−2.063 ± 0.059
Sudden unexpected death (Kotyńska et al. 2012)	4.01	1.370 ± 0.053	−2.290 ± 0.093
Fatal CO poisoning	3.98	1.190 ± 0.076	−2.216 ± 0.060

**Table 2:** Surface charge density and isoelectric point values for human thrombocytes (control, sudden unexpected death and fatal CO poisoning)

Groups	Isoelectric point	Surface charge density (10^−2^ C/m^2^)	
		At low pH values	At high pH values
Control (Kotyńska et al. 2012)	4.20	1.628 ± 0.206	−1.810 ± 0.185
Sudden unexpected death (Kotyńska et al. 2012)	2.90	0.213 ± 0.092	−2.802 ± 0.091
Fatal CO poisoning	3.81	1.000 ± 0.155	−2.355 ± 0.160

Biochemical profiles at autopsy may show considerable case variations due to various factors involving preexisting disorders, the cause of death, complications and environmental factors (Maeda et al. 2009). Luna (2009) postulated that forensic examiners need a real model of cadaver physiology to understand differences between the living and cadavers. One of the most important elements of this model is the evaluation of membrane changes in blood cells, as well as of different causes of death.

There are many problems in the diagnosis of postmortem changes in blood because of autolysis, putrefaction, artifacts during autopsy, analytical methodology and interpretation of results. In cadaver blood, like in the other tissue cells, blood cells lose their normal morphology and ability to be activated. Human erythrocytes had been extensively studied because of their relatively simple structure, ease of isolation and well-known methods of examination. For humans, the membrane integrity of erythrocytes is an important indicator of well-being. Chen and Cai (2006) observed postmortem membrane deformation of erythrocytes using atomic force microscopy, including shrinkage of cells, protuberances and fissures on cell membrane surfaces. In Halbhuber (1996) gamma-globulin receptors on the erythrocyte membrane were damasked due to changes in pH value. Nicák et al. (1999) observed erythrocyte deformability changes in human red blood cells under anoxic conditions using the method of cation-osmotic hemolysis.

The postmortem changes, based on many different processes, include alternations in membrane integrity. In erythrocytes, there are four main metabolic pathways (Embden-Meyerhof pathway, Rapaport-Luebering pathway, pentose phosphate pathway and methemoglobin reductase pathway), which are responsible for the normal function of hemoglobin and cell membrane integrity (Kasper et al. 2004). The increased positive and negative charges on the pH of the electrolyte solution (Fig. 1) are probably connected to disorders of these metabolic pathways, participating in the disintegration of the cell membrane.

CO poisoning distorts the oxygen–hemoglobin dissociation curve to the left and changes its sigmoidal shape toward a hyperbola. The decreased oxygen delivery is then sensed centrally, stimulating ventilatory efforts and increasing minute ventilation. The latter will increase uptake of CO, raise carboxyhemoglobin levels and result in a respiratory alkalosis. The clear effect is oxygen displacement and generation of carboxyhemoglobin, which is virtually unable to deliver oxygen to the tissues and is thereby responsible of various degrees of hypoxia (Blumenthal 2001). The tissue hypoxia that causes anaerobic metabolism and metabolic acidosis is often due to lactic acidosis. This process can lead to organ dysfunction, associated with cell death and tissue necrosis (Szczeklik 2006; Stryer 1995). CO produces tissue hypoxia by competing with oxygen for binding sites on the oxygen-carrying hemoproteins, including hemoglobin, myoglobin, cytochrome *c* oxidase and cytochrome P-450 (Thom 2009). CO increases cytosolic heme levels, leading to oxidative stress, and binds to platelet heme protein and cytochrome *c* oxidase, interrupting cellular respiration. When CO readily inhibits oxygen consumption by mitochondrial cytochrome *c* oxidase, it impairs electron transport in the electron transport chain, inhibits oxidative phosphorylation and leads to the formation of reactive oxygen species, which in turn to leads to cell membrane damage (Cronje et al. 2004; Thom et al. 2006; Neuman and Thom 2008). In our opinion the mechanism associated with cell membrane damage by reactive oxygen species could be responsible for the differences in isoelectric points and surface charge densities between the fatal CO poisoning and sudden unexpected death groups, observed for both erythrocytes and thrombocytes (Tables 1, 2).

The fatal CO poisoning as well as the sudden unexpected death groups showed increases in the positive electric charge of erythrocytes compared to the control group in the pH range 2–4. The increase in positive charge in our opinion may be associated with the appearance of new functional groups such as amino groups, derived from the disintegration of membrane proteins and/or lipids. However, the increase in negative charge at pH 4–11 may be associated with the increase of negatively charged functional groups, for example, carboxylic or phosphate, derived mainly from the decay of lipids and/or proteins. In conclusion, our studies indicate that, despite the disintegration of proteins and lipids, the integrity of the erythrocyte membrane is maintained. It could mean that fatal CO poisoning could determine postmortem changes of electric charge of human erythrocytes as well as sudden unexpected death.

Other important pathogenic effects of CO poisoning are the increased thrombotic tendency secondary to endothelial damage, increased platelet stickiness and alternations of the fibrinolytic pathway (Dileo et al. 2011). On the other hand, a mild inhibition of platelet aggregation after exposure to CO has also been reported (Chlopicki et al. 2006). In both examples the roles of CO intoxication in the mechanism of platelet aggregation, according to membrane changes, have not been completely described.

In thrombocytes, after fatal CO poisoning, we observed a reduction of positive and negative surface charges at whole pH values compared with the control group. As can be seen from Fig. 2, the experimental curves obtained for both fatal CO poisoning and sudden unexpected death show the same course in the pH 4–11 range. In our view, this means that there are no significant biological or chemical agents that could cause specific changes in thrombocyte membranes. However, the observed changes in the very narrow pH range of 2–4, which is an increase in the positive charge after CO poisoning compared to sudden unexpected death, may indicate the formation of new free negative functional groups, such as carboxyl, hydroxyl or phosphate groups.

Postmortem hypostasis should lead to an increase in the platelet count in cadaveric blood. Instead, the number of platelets actually decreased. Thomsen et al. (1999) showed that the decrease in the number of platelets in postmortem blood is not attributable to postmortem clotting but to a decrease in the number of countable platelets in postmortem blood. Postmortem platelets have lost the capacity to respond around 7–10 h after death and could not react with potentially activating substances in the subendothelial matrix. Parallel to this up to 10 h postmortem platelets in the blood of corpses are not activated, as shown by microscopic demonstration of the platelet activation markers CD62, CD63 and thrombospondin (Thomsen and Schmidtke 1997; Thomsen and Pueschel 1999).

## Conclusion

The effect of fatal CO poisoning on the surface charge of blood cells has been well characterized. Surface charge density values were determined from electrophoretic mobility measurements of blood cells performed at various pH levels. In blood cells, after fatal CO poisoning, changes of positive and negative surface charge at whole pH value compared with the control group were noted. The observed deviation may be caused by disregarding interactions between the functional groups of blood cells. From the other side, the immediate death can be responsible for slight changes in surface charge density in fatal CO poisoning because of the release of a small amount of reactive oxygen species, which damage the cell membrane to a moderate degree. However, our study is quite preliminary, and more in-depth research will be needed to define changes in surface charge density and to precisely estimate erythrocyte and thrombocyte cell membrane changes in CO poisoning, with various causes of death.
